# Application of iPSC to Modelling of Respiratory Diseases

**DOI:** 10.1007/5584_2019_430

**Published:** 2020

**Authors:** Ben A. Calvert, Amy L. Ryan (Firth)

**Affiliations:** Hastings Center for Pulmonary Research, Division of Pulmonary, Critical Care and Sleep Medicine, Department of Medicine, University of Southern California, Los Angeles, CA, USA; Hastings Center for Pulmonary Research, Division of Pulmonary, Critical Care and Sleep Medicine, Department of Medicine, University of Southern California, Los Angeles, CA, USA; Department of Stem Cell Biology and Regenerative Medicine, University of Southern California, Los Angeles, CA, USA

**Keywords:** Differentiation, Human models, iPSC, Lung disease, NKX2.1, Stem cell

## Abstract

Respiratory disease is one of the leading causes of morbidity and mortality world-wide with an increasing incidence as the aged population prevails. Many lung diseases are treated for symptomatic relief, with no cure available, indicating a critical need for novel therapeutic strategies. Such advances are hampered by a lack of understanding of how human lung pathologies initiate and progress. Research on human lung disease relies on the isolation of primary cells from explanted lungs or the use of immortalized cells, both are limited in their capacity to represent the genomic and phenotypic variability among the population. In an era where we are progressing toward precision medicine the use of patient specific induced pluripotent cells (iPSC) to generate models, where sufficient primary cells and tissues are scarce, has increased our capacity to understand human lung pathophysiology. Directed differentiation of iPSC toward lung presented the initial challenge to overcome in generating iPSC-derived lung epithelial cells. Since then major advances have been made in defining protocols to specify and isolate specific lung lineages, with the generation of airway spheroids and multi cellular organoids now possible. This technological advance has opened up our capacity for human lung research and prospects for autologous cell therapy. This chapter will focus on the application of iPSC to studying human lung disease.

## 1 Introduction

Respiratory disease is currently the third leading cause of morbidity and mortality worldwide (Lozano et al. 2012). It is also the leading cause of hospitalisations in developed countries (Hubbard 2006), placing huge individual and socioeconomic burdens on healthcare systems. Respiratory diseases encompass a wide range of disorders extending from more common diseases such as chronic obstructive pulmonary disease (COPD) and asthma to rare genetic disorders including cystic fibrosis (CF) and primary ciliary dyskinesia (PCD). Whilst each individual respiratory disorder possesses its own aetiology and pathophysiology, they often share many disease relevant commonalities, such as abnormal inflammation, increased susceptibility to infection and dysfunctional or damaged epithelia. Currently, many respiratory diseases are symptomatically managed with no effective treatment. Our understanding of disease initiation and progression is hindered through lack of robust *in vitro* models that closely reflect the disease phenotype as it occurs in humans for investigative research and drug screening. Many therapeutic “hits” discovered in mouse models do not translate successfully into humans leading to a high failure rate of lung therapeutics in clinical trials (Barnes et al. 2015). In this review, we evaluate the use of induced pluripotent stem cells (iPSC) for respiratory research and their potential for therapeutic applications in respiratory disease.

## 2 Induced Pluripotency

**Fig. 1:**
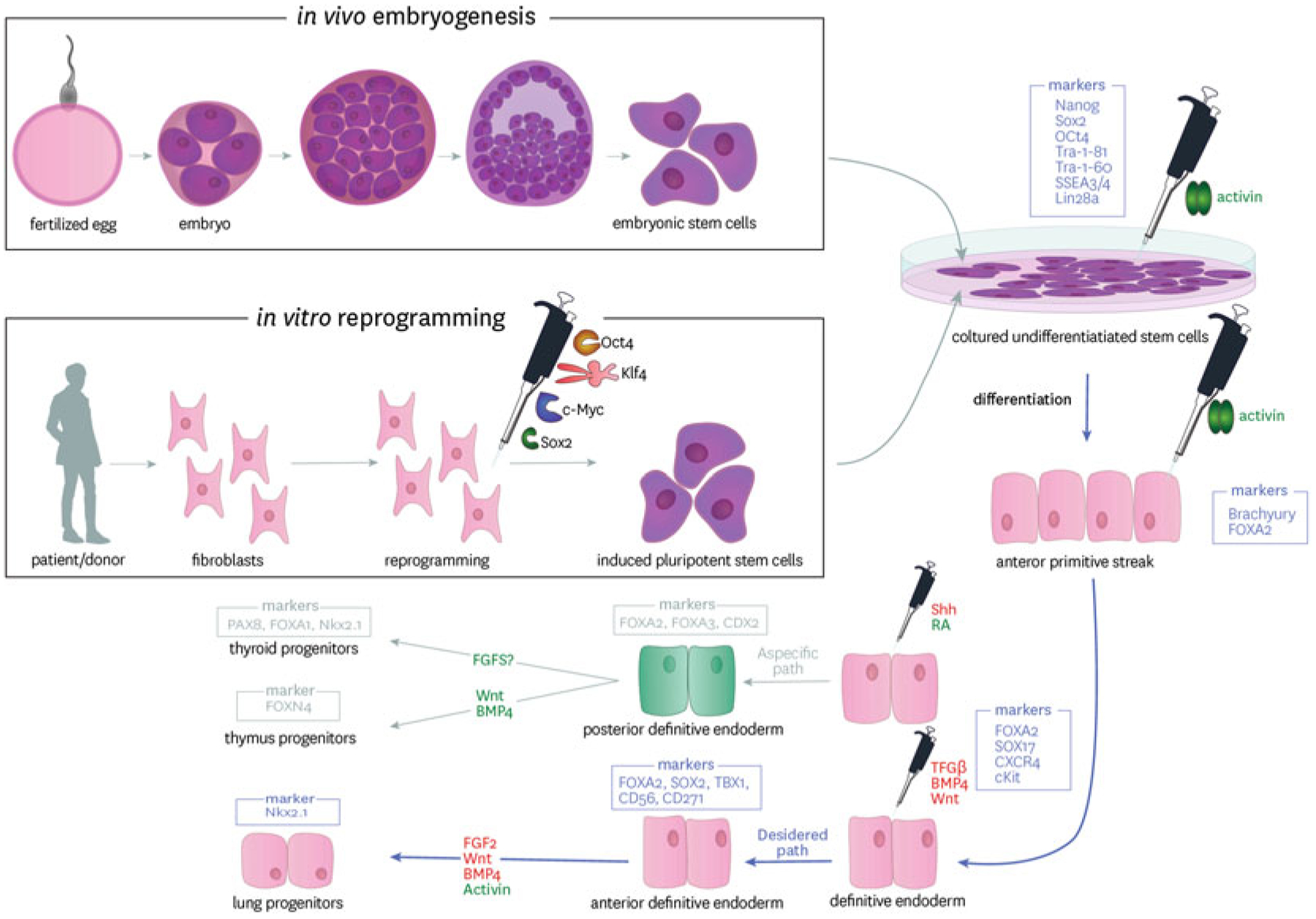
Pluripotent cell differentiation toward primordial lung progenitor cells. Pluripotent stem cells are isolated and expanded in vitro from the inner cell mass of the blastocyst (Embryonic Stem Cells or ESC) or from reprogramming of somatic cells from individuals (induced pluripotent stem cells or iPSC). Following a stepwize differentiation protocol mimicking the key steps in embryogenesis, cells are differentiated through FOXA2, SOX17 expressing definitive endoderm to anterior foregut endoderm and then NKX2.1 expressing primordial lung progenitors. The pipette symbol inidcates the cytokines and growth factors applied at each stage. The boxed genes represent key genes expressed at each stage. The red text indicates signalling that must be repressed and green text that must be activated

The discovery that fully differentiated/mature somatic cells can have pluripotency induced by Yamanaka et al. in 2006, ushered in a new era of genetic and cell biology research (Takahashi and Yamanaka 2006). This work identified that a minimal cocktail of 4 transcription factors, Oct4, Sox2, Klf4 and c-Myc, in combination with specific culture conditions was sufficient to reprogram terminally differentiated cells back into a state of pluripotency, akin to that of embryonic stem cells (ESCs) found in the inner cell mass of the blastocyst (Takahashi et al. 2007a, b; Okita et al. 2007) (Fig. 1). These cells acquired an infinite capacity for self-replication and differentiation into cells and tissues from all germ layers, including endodermal lung progenitors. As iPSC are generated by isolating cells from somatic tissues, they circumnavigate the ethical issues surrounding the use of ESCs (Murugan 2009). iPSC have revolutionized our capacity to carry out research in relevant human cells providing an exceptional tool for disease modelling, as well as possessing a huge potential for regenerative therapy.

Yamanaka and colleagues originally generated iPSC by transducing mouse fibroblasts with Oct4, Sox2, Klf4 and c-Myc transcription factors via pMX based retroviral vectors. Since then, other methods and factors have been utilized to successfully induce pluripotency in a wide range of somatic and germ line cells, these are summarized in Table 1. Initially, lentivirus became favoured over retroviruses due to its capability of infecting post-mitotic cells as well as dividing cells (Yamashita and Emerman 2006). Other virus types are also used, such as adenovirus and Sendai virus, favoured for their non-integrating nature (Zhou and Freed 2009), helping to maintain host genomic integrity with the original viral RNA diluted with each cell division (Fusaki et al. 2009). The most recent shift in technology is toward the use of non-viral methods of reprogramming including mRNAs (Warren et al. 2010), episomal plasmids (Okita et al. 2008), recombinant proteins (Zhou et al. 2009; Kim et al. 2009) using the four original Yamanaka factors. Other transcription factors have also found to be useful in the generation of iPSC. Many of these relate to the superfamilies of the transcription factors identified by Yamanaka, such as Oct3, Sox1 and Klf2 (Yu et al. 2007).

## 3 iPSC and Their Capacity for Disease Modelling

iPSCs have evolved rapidly as a technology, enabling the effective modelling of human disease, complimenting the more typical approaches using animal models and immortalised cell lines. Each model system has its own benefits and limitations (summarized in Table 2). While animal models of lung disease have substantially contributed to our knowledge of fundamental lung biology there has been little success in the translation of findings into the clinic for human use (Ma et al. 2018). Animal model studies of human diseases are often limited in the pathogenic aspects of the disease that they accurately recapitulate; for example, bleomycin instillation of animal models is often used to generate *in vivo* models of idiopathic pulmonary fibrosis, however does not accurately represent the onset or propagation of the disease. While informing us of some aspects, these models do not always replicate the complete aetiology and pathogenesis of the disease being studied. Primary isolated human cells are difficult to expand in culture without losing their phenotype with passage (Schiller and Bittner 1995). Further, human tissue availability can be limited and most often acquired post-mortem. This leads to a finite number of cells available for research from a limited patient population, which can result in limited use in high-throughput and drug screening research. Also, research into primary post-mitotic cells, such as that of neurones (Frade and Ovejero-Benito 2015), are restricted to the number of cells that can be initially isolated. This also limits the study of disease propagation and onset to what is typically an advanced disease state.

iPSCs provide an alternative and complimentary research tool that can overcome several limitations of animal models, primary and immortalized human cells. Restrictions of using primary and immortalized cell lines are surmounted due to their indefinite clonal expansion when maintained under specific culture conditions with the capacity for differentiation into multiple cell types comprising the human body (Kogut et al. 2014; Firth et al. 2015; Menon et al. 2015; Ward and Gilad 2019; Hnatiuk and Mercola 2019; Meijer et al. 2019; Fyfe 2019; Fiorotto et al. 2019; Hoshina et al. 2018; Mucci et al. 2018; Tan et al. 2018) including cells within the respiratory system. iPSC, therefore, have the potential to provide a seemingly unlimited source of patient/disease specific cells. This opens up multiple new options for research and the prospect for autologous cell therapy (Ebert et al. 2012). This chapter will focus on their application to studying human lung disease.

Genetic disorders are a prime example of where iPSC benefit over conventional *in vitro* disease modelling. Genetic diseases are often rare and have multiple subtypes, such as those seen in CF (Marson et al. 2016). Whilst the specific mutations of these subtypes are documented, access to patient specific material is limited, severely hindering studies of disease pathology. Instead, self-renewing iPSC can have the genetic mutation induced via state of the art gene editing technology, such as clustered regularly interspaced palindromic repeat (CRISPR)/Cas9 (Qi et al. 2013; Haurwitz et al. 2010; Wang et al. 2013; Cong et al. 2013). A concerted effort over the past decade has seen the evolution of protocols to differentiate iPSC to cells of the respiratory epithelium (Firth et al. 2014; Wong et al. 2012; Green et al. 2011; Cheng et al. 2012; Hawkins et al. 2017). This technology now enables rare genetic disorders to be modelled in a relevant and human cellular system. Several disease states have successfully been induced in iPSC including adenosine deaminase deficiency-related severe combined immunodeficiency (ADA-SCID), Shwachman-Bodian-Diamond syndrome (SBDS), Gaucher disease (GD) type III, Duchenne’s Muscular Dystrophy (DMD), Parkinson disease (PD), Huntington disease (HD), juvenile-onset, type 1 diabetes mellitus (JDM) (Park et al. 2008). The concept here is to develop an iPSC line and induce a disease phenotype in the cells by knocking out/in certain genes, or challenging the cells with factors that may onset disease. The field of neuroscience has particularly benefited from the use of iPSC (Wang and Doering 2012), as obtaining primary neuronal tissue is particularly challenging. Such diseases include Alzheimer’s disease (Kondo et al. 2013), Huntington’s disease (Kaye and Finkbeiner 2013) and schizophrenia (Brennand et al. 2011). The connexion to this paradigm is that iPSC derived from patient populations with rare genetic disorders, can be gene corrected through use of the same gene editing technology. While providing isogenic controls for *in vitro* evaluation this additionally opens up the potential for autologous cell based therapies reducing the need for immunosuppression and the likelihood of tissue rejection. In the respiratory field, proof-of–principle studies have demonstrated the correction of cystic fibrosis transmembrane regulator (CFTR) in CF patient derived iPSC, which were subsequently differentiated into functional epithelial cells (Firth et al. 2015; Crane et al. 2015). Providing a basis for novel iPSC based therapies for CF patients in the future.

## 4 Specification of Primordial Lung Progenitors from iPSC

Directed differentiation of iPSC toward lung endoderm presents its own set of challenges, which the field has made substantial progress toward elucidating over the past decade (Firth et al. 2014; Hannan et al. 2015; Wong and Rossant 2013). The lungs are a sophisticated organ system comprising of complex structures and over 40 different cell types; they include a complex vasculature, sympathetic and parasympathetic neuronal innovation, structural support and a specialized respiratory epithelium. To add to this complexity, the structure of the airways changes to meet its functional requirements along the proximal-distal axis. Similar to the gut, the respiratory system also contains a natural homeostatic microbiota, which can drastically alter during times of disease and stress (Dang and Marsland 2019; Man et al. 2017) and is an internal organ exposed to the exterior environment increasing the potential for epigenetic modification (Sakurada 2010; Hagood 2014). These features must all be considered when creating an *in vitro* model of respiratory disease and reflected in the advantages and limitations of any given model system. iPSC have the potential to investigate mechanisms of human lung development providing insights into the differentiation pathways from stem cell to fully differentiated tissues. In addition, they provide an opportunity to reverse a disease phenotype and investigate mechanisms of disease onset.

The first methods describing directed differentiation of the respiratory epithelium from iPSC focused primarily on specification of the lung endoderm (Cheng et al. 2012; Kadzik and Morrisey 2012; Longmire et al. 2012a). Subsequently, three pioneering papers were published differentiating cells to more mature cells in the respiratory epithelium (Wong et al. 2012; Kadzik and Morrisey 2012; Firth et al. 2014). These studies all strived to mimic lung embryonic development in a dish pushing cells through mesendoderm, to definitive endoderm (DE) followed by anteriorization of the ventral foregut endoderm (AFE) to primordial NKX2.1 expressing lung endodermal progenitor cells (LP). These cells have the capacity for differentiation into cells akin to that for the mature human lung including club, goblet, multiciliated, basal, alveolar and neuroendocrine cells.

Understanding lung development is critical to efficiently driving pluripotent cells to generate the cells and structures comprising the human lung. There is still a sparsity of specific knowledge of human lung development and much of our information is gained from transgenic mouse models tracing lineage specification (Bellusci et al. 1997a, b; Weaver et al. 1999, 2003; Okubo and Hogan 2004; Rawlins et al. 2009a, b). Lung organogenesis begins in the embryonic period with independent outpouchings of the ventral wall in the primitive foregut endoderm that elongate and branch into the surrounding mesenchyme. The respiratory mesenchyme is crucial in many developmental and homeostatic processes within the lung. It provides key signalling ligands to promote the development of lung structures, including alveolargenesis, airway branching and the vasculature. The mesenchyme is the primary source of transforming growth factor beta (TGFβ) in the developing lung (McCulley et al. 2015; White et al. 2006). It is an integral component for natural development and TGFβ knockout studies demonstrate impaired lung development (Sanford et al. 1997). The mesenchyme also provides the primary source of Wnt signalling, key for branching morphogenesis of airway epithelium (Miller et al. 2012) (Fig. 1).

**Fig. 2:**
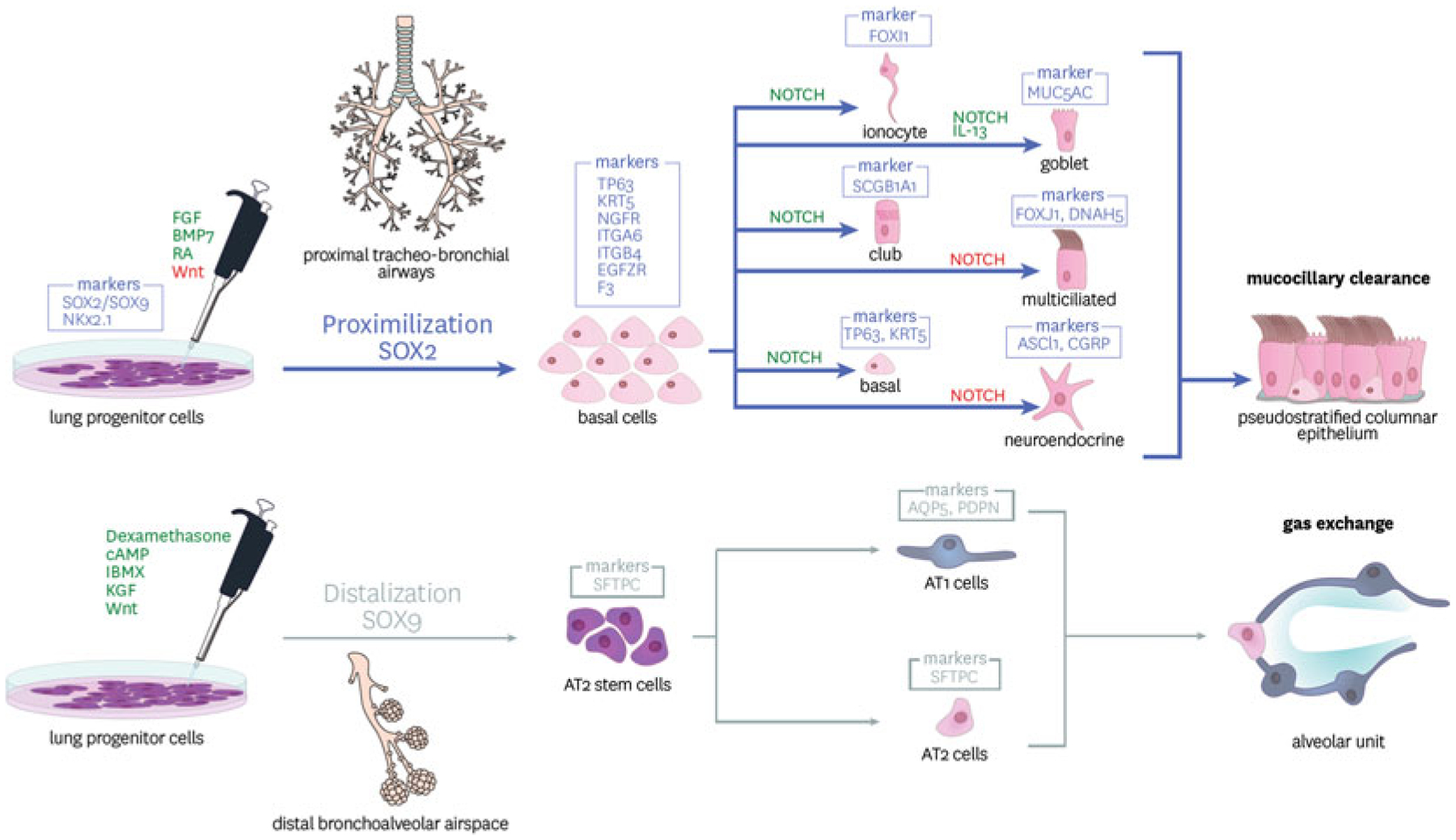
Differentiation of primordial lung progenitors towards proximal and distal lung fate. iPSC derived and purified lung progenitor cells expressing NKx2.1 can be directed toward proximal and distal fates through activation (green) and inhibition (red) of signalling pathways including those driven by FGFs, BMPs and wnts. The markers of the specific lineages are indicated in boxes above the cell types. Alveolar Type II (ATII) cells are the progenitor cells giving rise to mature ATII and ATI cells responsive for the functional alveolar unit for gas exchange. Sox2 expressing proximal basal cells are able to differentiate and give rise to all cells of the mature conducting airways including secretory, basal and multiciliated cells responsible for mucociliary clearance

DE gives rise to lungs, thyroid, pancreas, liver and intestines and is specified from the anterior primitive streak (APS), induced from pluripotent cells through strong activation of nodal and canonical wnt signaling, which are synergistically activated during gastrulation. This is mimicked in culture using Activin A and Wnt3a or Wnt agonist CHIR99021 (Kubo et al. 2004). The APS can be pushed to DE through persistent activation of nodal signalling and inhibition of bone morphogenetic protein (BMP), using DMH-1, to suppress mesoderm derivation (Green et al. 2011; Ogaki et al. 2013). Specification of DE from the mesendoderm has been optimized and results in a high efficiency of DE cells from iPSC (Mfopou et al. 2010). In embryonic development Wnt signalling plays an important role in many cellular functions, including differentiation and proliferation, *in Vitro* Wnt3A signalling is used to skew away from SOX2 expressing ectoderm and promote endodermal differentiation. DE also expresses cell surfaces markers CXCR4, a chemokine receptor important in cellular proliferation and cKit that can purify the DE through FACS sorting (Wong et al. 2010; Wang et al. 2012) (Fig. 2).

Anterioriziation of the DE to generate the foregut, identified through SOX2 and FOXA2 expressing cells does not appear to critically depend on Activin A/TGF-β-signalling. Inhibition of TGFβ is known to assist in driving AFE (Green et al. 2011) and an inhibition of Wnt- and BMP-signalling is critical in optimizing this transition. FOXA2 is an essential transcription factor for lung development, FOXA2−/− mice do not develop lungs (Wan et al. 2004; Aubin et al. 1997). Retinoic acid (RA), commonly used in lung differentiation protocols has dual effects and can either posteriorize or dorsalize the foregut creating PDX1-positive pancreatic duodenal cells. Its use in *in vitro* differentiation protocols is, therefore, not entirely clear. A number of studies have demonstrated the importance of RA, influencing micro RNAs, however, short pulses of RA can also maintain the stemness of iPSC through inhibition of the canonical Wnt pathway, essential for differentiation (De Angelis et al. 2018). In combination with Wnt/β-catenin, RA can act synergistically with FGF-2 and BMP-4 to generate CDX2-positive posterior endoderm further complicating the methods applied to iPSC differentiation (Davenport et al. 2016).

The successful generation of a primordial lung progenitor cell is accredited to the induction of lung transcription factor NKX2.1 (also known as TTF1) (Longmire et al. 2012a; Lazzaro et al. 1991). The function of NKX2.1 is not entirely understood. In mouse models, it is important in the development of respiratory tissue as well as other thoracic structures (Minoo et al. 1999; Minoo 2000). Purification of iPSC derived NKX2.1 primordial lung progenitor cells initially proved inefficient and purification was limited through lack of a suitable surface antigen. A recent study has shown that these NKX2.1 cells can be selected using a CD47-high, CD26-low surface marker expression profile (Hawkins et al. 2017). Alternatively, Carboxypeptidase M is also expressed in these cells and can be used to purify a similar population of lung progenitors (Konishi et al. 2016; Gotoh et al. 2014). While NKX2.1 defines specification of lung progenitor cells, it also has notable expression in the brain and thyroid tissues (Lee et al. 2001; Rossi et al. 1995; Acebron et al. 1995). Although the pathways that distinguish between these organ systems are not well-characterised, lineages can be identified though co-expression of PAX8 (thyroid, (Rossi et al. 1995) and PAX6 (forebrain, (Corbin et al. 2003; Takahashi and Osumi 2002).

Fibroblast Growth Factor (FGF) signalling is integral in defining lung endoderm and inducing NKX2.1 expression (Rankin and Zorn 2014; Dailey et al. 2005; Xian et al. 2005). In addition, sonic hedgehog (Shh) and transcriptional programs of the forkhead (Fox), and GATA-family members, are involved in specification of the lung from the AFE (Hogan 1999; Whitsett 1998). Differentiation towards lung progenitors can be directed away from specification of thyroid progenitors through controlled FGF2 expression. Studies have demonstrated that high concentrations of FGF activate Shh expression to generate NKX2–1 expressing lung progenitors and thyroid (Rankin and Zorn 2014; Serra et al. 2017; Kurmann et al. 2015; Longmire et al. 2012b). Dye et al. demonstrated that suppression of FGF activity whilst maintaining Shh signalling allowed for a more specific differentiation to lung primed NKX2.1 expressing cells (Dye et al. 2015). Sequential inhibition of TGFβ signalling, followed by subsequent activation of FGF and BMP4 signalling pathways can support further differentiation to lung epithelium (Longmire et al. 2012a).

## 5 Proximal and Distal Fate of Lung Progenitors

Lung buds arise from the lateral part of the foregut prior to forming the trachea and recent data suggests that the progenitors at the leading tip of these lung buds differ in humans and mice and can specify both the proximal and distal regions of the lung (Miller et al. 2018; Danopoulos et al. 2018; Nikolic et al. 2017). To study these fate decisions in humans a three-dimensional organoid system has been established to culture fetal lung bud tips (Miller et al. 2018; Danopoulos et al. 2018; Nikolic et al. 2017). Cells at the leading tip of these buds express NKx2.1, SOX2 and SOX9 in humans; this contrasts with the cells in the same region of the mouse which either co-express NKX2.1 and SOX2 or SOX9 (Miller et al. 2018). A similar population of cells has been observed in iPSC derived lung progenitors from humans (Miller et al. 2018).

Detailed analysis of the regulation of proximal and distal fate decisions has been extensively studied in mice using lineage tracing models (Rawlins et al. 2009b; Barkauskas et al. 2013; Rock et al. 2009). In humans, we rely on the development of robust *in vitro* models. Both fetal and iPSC derived lung bud organoids will differentiate when exposed to FGF7, CHIR and Retinoic Acid, generating cells akin to those in the human airways (Miller et al. 2018). By utilizing more sophisticated scaffold materials, tubular airway-like structures can also be replicated *in vitro*, resembling that of the canalicular development in lung embryogenesis (Dye et al. 2016). Additional factors to consider when developing more complex models, is the importance of the mesenchymal supporting cells in controlling the fate of lung progenitors towards a proximal or distal epithelial phenotypes. Signals between the mesenchyme and epithelium are critical in lung development, and supplying the exogenous growth factors to an *in vitro* system may be in sufficient to allow us to fully appreciate the signals responsible for proximal and distal human lung fate decisions (El Agha et al. 2014).

## 6 Tracheo-Bronchial Differentiation and Disease Models

Specification of distal and proximal lung cells requires precise spatiotemporal regulation of Wnt, Notch and FGF signaling pathways. The proximal airways, comprising of tracheal and bronchial cartilaginous airways, are populated by basal cells, as the predominant progenitor cell, in addition to club and goblet secretory cells and multiciliated cells as the primary functional epithelium. SOX2 expression delineates the proximal airways from their distal counterparts that continue to selectively express SOX9 (Danopoulos et al. 2018). As discussed above, bud tip progenitors co-express both SOX2 and SOX9 during psuedoglandular phase of human embryogenetic development, however, will become determined before development reaches the canalicular stages, controlled by signals received within their proximity microenvironment (Danopoulos et al. 2018).

Currently there are no published studies specifically focusing on the specification and expansion of an iPSC-derived basal cell. Proximal airway basal cells are currently identified by the expression of cytokeratin 5 (KRT5), TP63, nerve growth factor receptor (NGFR) and integrin alpha 6 (ITGA6 or CD49f) (Daniely et al. 2004). During development, it appears that SMAD signaling plays a role in the lineage differentiation pushing away from lung progenitor stem cells. TGFβ and BMP4 mediated SMAD signaling has, however, been demonstrated to promote the differentiation from bud tips to a basal cell like phenotype, using an organoid based *in vitro* system (Dye et al. 2015). At this stage, SMAD inhibition promotes the maintenance of the basal cell phenotype and further differentiation beyond a precursor cell (Mou et al. 2016).

Another key component for differentiation to lung basal cell epithelium is NOTCH signaling (Rock et al. 2011). Activation of Notch signaling pathways is critical in embryonic development and plays various roles in a more developed system. In the basal cell, NOTCH signaling is involved in its further differentiation to a mature epithelial subtype. Maturation of airways cells is most commonly achieved at an “air-liquid interface” or ALI, a platform arguably more complex than *in vitro* systems for most other organ systems (de Jong et al. 1994). In this system, human bronchial epithelial cells (HBEC) are seeded to transwell inserts, allowed to grow to confluence generating an epithelial barrier with tight junctions. Once sufficient trans-epithelial electrical resistance (TEER) is generated, the apical media is removed generating an apical air interface. Over a 28-day period the cells undergo process of polarization, pseudo stratification and maturation comprising predominantly of basal, secretory (goblet and club cells) and multiciliated cells. Inhibition of NOTCH promotes a basal cell to ciliated cell transition, whist continued activation of NOTCH pathways promote a secretory fate (Rock et al. 2011). Successful differentiation of ALI cultures from iPSC was demonstrated in some of the first protocols published (Wong and Rossant 2013; Firth et al. 2014). Since than several other laboratories have generated pseudostratified epithelium reflecting that of the primary airway cell differentiation in vitro (Konishi et al. 2016; McCauley et al. 2017; Huang et al. 2015).

## 7 Distal Lung and Alveolar Differentiation and Disease Models

Cells expressing NKX2–1 stand as the distinct lung progenitor that may differentiate into any lung cellular phenotype. Lung patterning during embryogenesis requires determination to distinguish the saccular generation of the distal alveolar spaces. *In vitro* generation of more distally aligned airway cells can be controlled via Wnt signalling. It had been demonstrated that high Wnt activation could generate alveolar progenitors whilst conversely supressed Wnt signalling generated more proximal cell types (McCauley et al. 2017). This is primarily achieved by culturing the cells in the presence of a potent GSK3β inhibitor known as CHIR99021. Inhibiting the ability of this enzyme to activate Wnt and its downstream machinery. The result is the robust generation of distal/alveolar epithelial cells (Jacob et al. 2017).

Alveolar epithelium is comprised of two major subtypes; alveolar type 1 (AT1) & alveolar type 2 (AT2) cells. In short, AT1 cells provide the structural basis of the alveolar spaces and primary function for gaseous exchange, whilst AT2 cells are primarily secretory and provide a supporting role to the AT1 cells. However, there is distinct multifunctional heterogeneity within these cell types. This was eloquently demonstrated utilising a surfactant protein C (SPC) (AT2 specific marker) reporter line, whereby phenotypic profiling multiple subtypes within this specific cell population (Lee et al. 2013). Interestingly, mutations in SPC are known to cause interstitial lung disease, and have been modelled utilising iPSC derived AT2 cells (Jacob et al. 2017). The AT2 cells derived from iPSCs are found to be NKX2.1 and closely resemble that of the foetal lung AT2 cells, based on a genetic profiling. This model is now being utilised to study the influence of 173 T SPC mutation and effectively model interstitial lung disease *in vitro*. Other distally aligned respiratory diseases have also been modelled *in* vitro utilising iPSC-based techniques. These include IPF models where 3-D organoids have been able to replicate disease characteristics including accumulation of extracellular matrix and mesenchymal cells, suggesting the potential for modelling fibrotic lung disease *in vitro* (Wilkinson et al. 2017; Strikoudis et al. 2019).

The development of lung progenitor cells is limited by our understanding of the phenotype and function of their primary counterpart. To date no direct comparison has been made between iPSC-derived cells and *in vitro* cultured primary cells. With substantial profiling of cells in progress through programs such as LungMAP (https://lungmap.net), it is hoped that a more extensive profile of basal cells and potential sub populations of progenitor cells will lead to increased options of specific cellular surface markers for specific identification and isolation of the definitive stem cells.

## 8 iPSC and their Capacity for Tissue Regeneration

Many degenerative disorders, such as COPD, do not have effective disease modifying treatments. In theory, iPSC could be generated from each patient diseases, differentiated to the relevant stem/progenitor cell and engrafted back into the patient’s diseased and damaged lung. Furthermore, genetic disorders, such CF and primary ciliary dyskinesia, could be corrected using state-of-the-art gene editing technologies prior to engraftment. Published data has demonstrated the successful use of iPSC-derived cells and their regenerative therapeutic potential in a multitude of disorders using animal models and *in vitro* based techniques. These include models of liver injury (Liu et al. 2011), muscular related disorders (Kazuki et al. 2010; Tan et al. 2012), blood/immunological disorders (Suzuki et al. 2013), cardiovascular disease (Shiba et al. 2016) and spinal cord injury (Nori et al. 2011), among others (Li et al. 2017). In the clinic, patient specific, iPSC-derived retinal epithelial cells have successfully been transplanted back into patients with macular degeneration, marking the first attempt of iPSC to treat a patient population (Mandai et al. 2017). Unfortunately, the reality of this is infinitely more challenging and complex for the lung. The lung comprises of over 40 different functional cell types forming airways, vasculature, cartilage, immune system, sympathetic and parasympathetic neural tissues, glands and supportive parenchyma. In the case of lung disease, it unlikely that one cell type is affected in isolation and more reasonable to think of changes more globally with specific microenvironments adapting to maximize protection and function of the lung for gas exchange. Long-term replacement of cells will likely require access to the relevant cellular niche for long-term reconstitution and adaptation of the niche to reflect that of a non-diseased lung to sustain a “healthy” engrafted cell and derivatives. Progress in the field of lung regeneration has been substantial but we now need to start thinking toward more complex models, which more closely recapitulate the *in vivo* cellular niche. This will require, at minimum, collaborative efforts between biologists, bioengineers and novel translational imaging techniques.

## 9 The Future of iPSC for Respiratory Disease

iPSC present a novel, human and patient specific avenue for research and therapeutic advancement. iPSC have enabled the generation of human and disease-related models to be created in vitro providing an unprecedented access to human biology. Like any model system, they are not without their limitations and should be used alongside other available systems suitable for completely addressing the specific experimental question at hand. For the lung, exposure of the cells to the environment, a constant for the millieu of cells in the airways, is not yet considered in this model system. Furthermore, epigenetic changes causative of disease are likely wiped during the reprogramming process loosing these markers as disease phenotypes. However, by utilising iPSC, a seemingly limitless source of cells is available and representative of the parent genetic profile enabling both human and patient specific cellular models to be developed. Further, the use of iPSC allows for robust investigations into the developmental pathways involved in a human cell-based system that would otherwise be challenging. In iPSC, individual genes can be manipulated and evaluated side-by-side with their isogenic counterparts enabling precise effects of specific genes to be evaluated. As such, iPSC provide an incredibly valuable model system as we progress to an era of personalized medicine.

## 10 Concluding Remarks

Substantial progress has been made since Yamanaka’s discovery of induced pluripotency in humans in 2007. From initially identifying an iPSC inducing minimal cocktail of transcription factors, to sucessful use of iPSC as a therapeutic tool, highlights the speed at which this technlogy has evolved. Although initial derivation of relevant respiratory cells from iPSC lagged behing other organ systems, we still have a plethora of methods available to study respiratory disease in a biologically relevant cell type, overcoming the shortfall of current model systems. Progress is currently limited by our fundamental lack of understanding of the mechanisms controlling human lung development, the precise identity and function of human lung cell types and the genetic and epigenetic control of human lung fate. As our capacity to model human lung disease evolves, so will our understanding of the pathogenesis of human lung disease. iPSC models remain an exciting prospect.

## Figures and Tables

**Table 1:** Methods for reprogramming somatic cells to iPSC

Method	Vector	Genomic integration	Advantages	Disadvantages	References
Viral	Lentivirus, retrovirus	Integrating	High efficiency stable expression can be inducible	Tendency for insertional mutagenesis	(Takahashi et al. (2007b), Yu et al. (2007), Ohnuki et al. (2014), Carey et al. (2009), Hotta et al. (2009), Sommer et al. (2009) and Liao et al. (2008)
Viral	Sendai, adenovirus	Non-integrating	High efficiency	Tendency to carry host genome	Zhou and Freed (2009), Fusaki et al. (2009), Fujie et al. (2014) and Suzuki et al. (2008)
			Non-integrating		
Non-viral	Episomal vectors	Non-integrating	Virus free	Lower efficiency	Okita et al. (2011), Yu et al. (2009) and Hu and Slukvin (2013)
			Single transfection		
Non-viral	PiggyBac transposon	Non-integrating	Evidence for more rapid reprogramming	Labour intensive and relatively low efficiency	Yusa et al. (2009) and Stadtfeld and Hochedlinger (2009)
				Inefficient excision	
Non-viral	Mini-circle vectors	Non-integrating	Virus free. Higher efficiency of transfection	Longer ectopic expression	Narsinh et al. (2011) and Jia et al. (2010)
Non-viral	Plasmid	Non-integrating	Virus free	Low efficiency	Kim et al. (2016), Dowey et al. (2012), Karow et al. (2011), Si-Tayeb et al. (2010) and Okita et al. (2010)
				Multiple rounds of transfection	
Non-viral	Protein	Non-integrating	No genetic material, direct protein delivery	Very slow reprogramming kinetics, very low efficiency	Tammam et al. (2016), Nemes et al. (2014), Thier et al. (2012) and Thier et al. (2010)

**Table 2:** Possible iPSC derived models for lung disease

Model	Species	Model usage	Benefits	Limitations	References
Organoid	Human	Lung structural development	Multiple cell types, spatially organized 3D system	Unsuitable for specific pathway analysis. No air interface	Dye et al. (2015), Wilkinson et al. (2018) and Chen et al. (2017)
Air liquid Interface	Human mouse	Epithelial barrier formation and function	Physiologically relevant air interfacing system, high throughput potential, TEER measurement	No presence of mesenchymal niche cells	Firth et al. (2014), Wong et al. (2012) and Hawkins et al. (2017)
Transplant	Human mouse	Cell engraftment and in vivo regeneration	Study engraftment potential of cell-based therapy, In vivo niche	Long-term human studies lacking, immune suppression	Shafa et al. (2018) and Okuyama et al. (2019)
Spheroid	Human mouse	Cellular and structural modelling, functional assays	Suitable for stringent pathway analysis, functional swelling	No air interface, usually lacks niche cells	Konishi et al. (2016), Gotoh et al. (2014), Dye et al. (2015) and Jacob et al. (2017)

*TEER* Trans Epithelial Electrical Resistance

## Abbreviations

ADA-SCID: adenosine deaminase deficiency-related severe combined immunodeficiency
AFE: anteriorization of the ventral foregut endoderm
ALI: air-liquid interface
APS: anterior primitive streak
BMP: bone morphogenetic protein
CDX2: caudal type homeobox 2
CF: cystic fibrosis
CFTR: cystic fibrosis transmembrane regulator
COPD: chronic obstructive pulmonary disease
CXCR4: C-X-C chemokine receptor type 4
DE: definitive endoderm
DMD: Duchenne’s Muscular Dystrophy
DMH-1: dorsomorphin homolog 1
ESC: embryonic stem cells
FACS: flow activated cell sorting
FGF-2: fibroblast growth factor 2
FOXA2: forkhead box A2
GATA: GATA binding protein
GD: Gaucher disease
HBEC: human bronchial epithelial cells
HD: Huntington disease
IPF: idiopathic pulmonary fibrosis
iPSC: induced pluripotent stem cells
ITGA6 or CD49f: integrin alpha 6
JDM: juvenile-onset, type 1 diabetes mellitus
KLF4: Kruppel like factor 4
KRT5: cytokeratin 5
LP: lung endodermal progenitor cells
mRNAs: messenger ribonucleic acid
NGFR: nerve growth factor receptor
OCT4/POU5F1: domain, class 5, transcription factor 1
PAX6: Paired box gene 6
PAX8: Paired box gene 8
PCD: primary ciliary dyskinesia
PD: Parkinsons disease
PDX1: pancreatic and duodenal homeobox 1
RA: Retinoic acid
RNA: ribonucleic acid
SBDS: Shwachman-Bodian-Diamond syndrome
Shh: Sonic hedgehog
SOX2: sex determining region Y 2
SPC: surfactant protein C
TEER: trans-epithelial electrical resistance
TGFβ: transforming growth factor beta
TP63: tumor protein p63
TTF1: thyroid transcription factor 1
Wnt: wingless INP pathway
